# Methicillin-Resistant *Staphylococcus aureus* in Retail Meat, Detroit, Michigan, USA

**DOI:** 10.3201/eid1706.101095

**Published:** 2011-06

**Authors:** Kanika Bhargava, Xiaogang Wang, Susan Donabedian, Marcus Zervos, Liziane da Rocha, Yifan Zhang

**Affiliations:** Author affiliations: Wayne State University, Detroit, Michigan, USA (K. Bhargava, X. Wang, L. da Rocha, Y. Zhang); Henry Ford Health Systems, Detroit (S. Donabedian, M. Zervos)

**Keywords:** MRSA, USA300, spa, meat, poultry, Staphylococcus aureus, antimicrobial drug resistance, Michigan, bacteria, letter

**To the Editor:** Because methicillin-resistant *Staphylococcus aureus* (MRSA) has been identified in retail meat worldwide (*1**–**4*), the potential exists for its transmission to humans. Of the various meat products surveyed, pork had the highest contamination rate in the United States and Canada (*1**,**2*), as did beef in South Korea (*3*) and poultry in the Netherlands (*4*). The study in South Korea also observed MRSA from chicken, which demonstrated sequence type (ST) 692 by multilocus sequence typing (MLST), a type distinct from that isolated in beef and pork. Despite sample size variations, these studies suggested that MRSA contamination in different meat categories can vary by location and that molecular distinction may exist among MRSA isolates in meat of different origin.

We collected 289 raw meat samples (156 beef, 76 chicken, and 57 turkey) from 30 grocery stores in Detroit, Michigan, USA, during August 2009–January 2010. Up to 3 presumptive *S. aureus* colonies per sample were identified by coagulase test and species-specific PCR (*1*). Antimicrobial drug MICs were determined and interpreted according to Clinical and Laboratory Standards Institute guidelines (*5*). *S. aureus* were characterized by pulsed-field gel electrophoresis (PFGE), *mecA* identification, staphylococcal cassette chromosome (SCC) *mec* typing, Panton-Valentine leukocidin identification, *agr* typing, MLST, and *spa* typing as described (*1**,**6*).

Sixty-five (22.5%) samples yielded *S. aureus*: 32 beef (20.5%), 19 chicken (25.0%), and 14 turkey (24.6%) samples. Six samples, consisting of 2 beef (1.3%), 3 chickens (3.9%), and 1 turkey (1.7%), were positive for MRSA as evidenced by the presence of *mecA*. The overall lower prevalence of *S. aureus* and MRSA than that found in a previous study in the United States (40% and 5%, respectively) (*1*) might be explained by our exclusion of pork because pork and swine production have been major reservoirs of MRSA (*4**,**7*). However, different geographic location and cold sampling seasons in this study also might have caused the variations. The only multidrug-resistant MRSA isolate in this study (MRSA1) was from beef and was resistant to β-lactams, macrolides, and fluoroquinolones (Figure).

**Figure:**
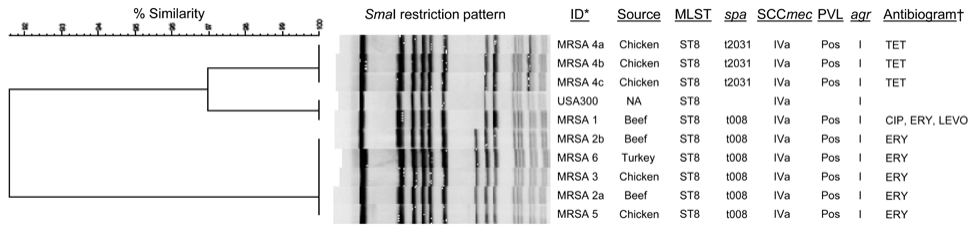
Dendrogram showing comparison of *Sma*I pulsed-field gel electrophoresis patterns, staphylococcal cassette chromosome (SCC) *mec* type, Panton-Valentine leukocidin (PVL) content, and *agr* type of methicillin-resistant *Staphylococcus aureus* (MRSA) isolated from meat samples. All MRSA isolates were resistant to β-lactam antimicrobial drugs (ampicillin, penicillin, and oxacillin) and grew on the 6 µg/mL of cefoxitin for screening methicillin resistance. *Isolates with the same arabic numbers were from the same sample; †only resistance to non–β-lactam antimicrobial drugs was listed. ID, identification; MLST, multilocus sequence typing; ST, sequence type; pos, positive; TET, tetracycline; NA, not available; CIP, ciprofloxacin; ERY, erythromycin; LEVO, levofloxacin.

Although an extra band was generated in MRSA 2a, 2b, 3, 5, and 6 by PFGE, all 9 MRSA isolates belonged to USA300 (Figure). Multiple isolates from the same samples (MRSA 2a and 2b; MRSA 4a, 4b, and 4c) demonstrated indistinguishable PFGE patterns and other characteristics, which suggested identical MRSA clones. Moreover, MLST, SCC*mec* typing, *agr* typing, and *pvl* detection showed all strains to be positive for ST8, SCC*mec* IVa, *agr* I, and Panton-Valentine leukocidin, which are typical characteristics of USA300 clones. However, *spa* typing identified 2 distinct *spa* types, t008 (11–19–12–**21–17**–34–24–34–22–25) and t2031 (11–19–12–**12–34–**34–24–34–22–25) (repeat variants in **boldface**), which differed by 5 nucleotides. t008, the most common *spa* type of USA300, was identified in 6 isolates of beef, chicken, and turkey origin, whereas t2031 was recovered from MRSA4a, 4b, and 4c from a chicken sample. The nucleotide variation in t2031 caused amino acid changes from glycine-asparagine in t008 to asparagine-lysine. The single nucleotide difference between repeats 12 (GGT) and 21 (GGC) and repeats 34 (AAA) and 17 (AAG) resulted in no amino acid change, with glycine and lysine encoded, respectively.

Unlike studies in Europe, where researchers have reported the animal MRSA clone ST398 from various meat products (*4*), all MRSA isolates in our study were USA300, which suggests a possible human source of contamination during meat processing (*1*). The failure to identify ST398 in the US retail meat also indicates that the human MRSA clones might be better adapted in meat processing than ST398 in this country. Since ST398 is widespread in animals and meat in Europe and has been isolated from other parts of the world (*8*), it is not too bold to predict that ST398 might appear in US meat in the future, especially after the recent report of ST398 from US swine (*7*).

The 5-nt difference between t2031 and t008 implicates multiple MRSA clones in poultry. Previous studies have shown *spa* variants of USA300 from clinical cases associated with distinctive symptoms (*9**,**10*). A single repeat variant, t024, showed substantial genetic, epidemiologic, and clinical differences from t008 in Denmark (*10*). Researchers in Japan also recovered 2 *spa* variants of USA300: t024, which causes blood infections, and t711, which is associated with subcutaneous abscesses (*9*). In both studies, t024 behaved as hospital-associated MRSA, suggesting that *spa* variants of USA300 could lead to different clinical outcomes. Therefore, we can reasonably assume that variants with a meat origin also might have different public health implications; further research on their virulence potential would be helpful to elucidate this possibility.

Despite the recovery of MRSA from retail chicken and t2031 that has an antibiogram distinct from t008, except for β-lactam resistance, several questions remain about whether more *spa* variants are present in poultry (or meat). These include whether t2031 is more adaptable to chicken production because of the 2 amino acid difference from t008, or whether t2031 is linked with specific antimicrobial drug resistance phenotypes other than β-lactam resistance.
